# Supplementary material for: Cost-effectiveness analysis of dapagliflozin for people with chronic kidney disease in Malaysia
Source: PLoS One. 2024 Mar 6;19(3):e0296067. doi: 10.1371/journal.pone.0296067 (PMC10917287; doi:10.1371/journal.pone.0296067)
Supplement: S1 Table — (DOCX) [file pone.0296067.s002.docx]

Supplementary Table 1. CKD transition matrix - Dapagliflozin + SoC - Mean (SE)

| **Mean (SE)** | | **To** | | | | | | | | **Reference** |
| --- | --- | --- | --- | --- | --- | --- | --- | --- | --- | --- |
|  |  | **CKD 1** | **CKD 2** | **CKD 3a** | **CKD 3b** | **CKD 4** | **CKD 5** | **Dialysis** | **Kidney transplant** |  |
| **Months 0-4** | | | | | | | | | | |
| From | CKD 1 | 0.586 (0.076) | 0.219 (0.064) | 0.049 (0.033) | 0.049 (0.033) | 0.024 (0.024) | 0.024 (0.024) | 0.024 (0.024) | 0.025 (0.024) | DAPA-CKD[37] |
|  | CKD 2 | 0.018 (0.005) | 0.709 (0.016) | 0.246 (0.015) | 0.019 (0.005) | 0.003 (0.002) | 0.003 (0.002) | 0.001 (0.001) | 0.001 (0.001) |  |
|  | CKD 3a | 0.001 (0.001) | 0.079 (0.006) | 0.749 (0.009) | 0.162 (0.008) | 0.008 (0.002) | 0.000 (0.000) | 0.000 (0.000) | 0.000 (0.000) |  |
|  | CKD 3b | 0.001 (0.000) | 0.005 (0.001) | 0.079 (0.004) | 0.812 (0.006) | 0.102 (0.005) | 0.001 (0.000) | 0.000 (0.000) | 0.000 (0.000) |  |
|  | CKD 4 | 0.001 (0.001) | 0.003 (0.001) | 0.006 (0.002) | 0.143 (0.008) | 0.843 (0.008) | 0.004 (0.001) | 0.001 (0.001) | 0.001 (0.000) |  |
|  | CKD 5 | 0.001 (0.001) | 0.002 (0.001) | 0.002 (0.001) | 0.001 (0.001) | 0.027 (0.005) | 0.920 (0.008) | 0.045 (0.006) | 0.002 (0.001) |  |
|  | Dialysis | 0.000 (0.000) | 0.000 (0.000) | 0.000 (0.000) | 0.000 (0.000) | 0.000 (0.000) | 0.000 (0.000) | 0.998 (0.000) | 0.002 (0.000) | National Dialysis and Transplant Registry MDTR[38] |
|  | Kidney transplant | 0.000 (0.000) | 0.000 (0.000) | 0.000 (0.000) | 0.000 (0.000) | 0.000 (0.000) | 0.000 (0.000) | 0.03 (0.001) | 0.943 (0.001) |  |
| **Months 5 and onwards** | | | | | | | | | | |
| From | CKD 1 | 0.891 (0.017) | 0.070 (0.014) | 0.009 (0.005) | 0.015 (0.007) | 0.006 (0.004) | 0.003 (0.003) | 0.003 (0.003) | 0.003 (0.003) | DAPA-CKD[37] |
|  | CKD 2 | 0.005 (0.001) | 0.909 (0.004) | 0.078 (0.004) | 0.006 (0.001) | 0.002 (0.001) | 0.000 (0.000) | 0.000 (0.000) | 0.000 (0.000) |  |
|  | CKD 3a | 0.001 (0.000) | 0.025 (0.001) | 0.913 (0.003) | 0.059 (0.002) | 0.002 (0.000) | 0.000 (0.000) | 0.000 (0.000) | 0.000 (0.000) |  |
|  | CKD 3b | 0.000 (0.000) | 0.001 (0.000) | 0.025 (0.001) | 0.938 (0.002) | 0.035 (0.001) | 0.000 (0.000) | 0.000 (0.000) | 0.000 (0.000) |  |
|  | CKD 4 | 0.000 (0.000) | 0.000 (0.000) | 0.001 (0.000) | 0.035 (0.002) | 0.952 (0.002) | 0.010 (0.001) | 0.001 (0.000) | 0.000 (0.000) |  |
|  | CKD 5 | 0.001 (0.001) | 0.002 (0.001) | 0.002 (0.001) | 0.001 (0.001) | 0.027 (0.005) | 0.920 (0.008) | 0.045 (0.006) | 0.002 (0.001) |  |
|  | Dialysis | 0.000 (0.000) | 0.000 (0.000) | 0.000 (0.000) | 0.000 (0.000) | 0.000 (0.000) | 0.000 (0.000) | 0.998 (0.000) | 0.002 (0.000) | National Dialysis and Transplant Registry MDTR[38] |
|  | Kidney transplant | 0.000 (0.000) | 0.000 (0.000) | 0.000 (0.000) | 0.000 (0.000) | 0.000 (0.000) | 0.000 (0.000) | 0.03 (0.001) | 0.943 (0.001) |  |
