# Supplementary material for: Cost-effectiveness analysis of dapagliflozin for people with chronic kidney disease in Malaysia
Source: PLoS One. 2024 Mar 6;19(3):e0296067. doi: 10.1371/journal.pone.0296067 (PMC10917287; doi:10.1371/journal.pone.0296067)
Supplement: S2 Table — (DOCX) [file pone.0296067.s003.docx]

Supplementary Table 2. CKD transition matrix – Placebo + SoC – Mean (SE)

| **Mean (SE)** | | **To** | | | | | | | | **Reference** |
| --- | --- | --- | --- | --- | --- | --- | --- | --- | --- | --- |
|  |  | **CKD 1** | **CKD 2** | **CKD 3a** | **CKD 3b** | **CKD 4** | **CKD 5** | **Dialysis** | **Kidney transplant** |  |
| **Months 0-4** | | | | | | | | | | |
| From | CKD 1 | 0.375 (0.084) | 0.313 (0.081) | 0.156 (0.064) | 0.031 (0.030) | 0.031 (0.030) | 0.031 (0.030) | 0.031 (0.030) | 0.031 (0.030) | DAPA-CKD[37] |
|  | CKD 2 | 0.009 (0.003) | 0.770 (0.014) | 0.195 (0.013) | 0.016 (0.004) | 0.004 (0.002) | 0.002 (0.002) | 0.002 (0.002) | 0.001 (0.001) |  |
|  | CKD 3a | 0.002 (0.001) | 0.070 (0.005) | 0.774 (0.009) | 0.149 (0.007) | 0.004 (0.001) | 0.000 (0.000) | 0.000 (0.000) | 0.000 (0.000) |  |
|  | CKD 3b | 0.002 (0.001) | 0.004 (0.001) | 0.084 (0.005) | 0.826 (0.006) | 0.082 (0.005) | 0.001 (0.001) | 0.001 (0.000) | 0.000 (0.000) |  |
|  | CKD 4 | 0.001 (0.001) | 0.002 (0.001) | 0.005 (0.002) | 0.127 (0.008) | 0.856 (0.009) | 0.007 (0.002) | 0.001 (0.001) | 0.001 (0.001) |  |
|  | CKD 5 | 0.001 (0.001) | 0.001 (0.001) | 0.001 (0.001) | 0.002 (0.001) | 0.038 (0.005) | 0.910 (0.008) | 0.044 (0.005) | 0.003 (0.002) |  |
|  | Dialysis | 0.000 (0.000) | 0.000 (0.000) | 0.000 (0.000) | 0.000 (0.000) | 0.000 (0.000) | 0.000 (0.000) | 0.998 (0.001) | 0.002 (0.001) | National Dialysis and Transplant Registry MDTR[38] |
|  | Kidney transplant | 0.000 (0.000) | 0.000 (0.000) | 0.000 (0.000) | 0.000 (0.000) | 0.000 (0.000) | 0.000 (0.000) | 0.03 (0.001) | 0.943 (0.001) |  |
| **Months 5 and onwards** | | | | | | | | | | |
| From | CKD 1 | 0.884 (0.020) | 0.075 (0.016) | 0.015 (0.007) | 0.011 (0.006) | 0.004 (0.004) | 0.004 (0.004) | 0.004 (0.004) | 0.004 (0.004) | DAPA-CKD[37] |
|  | CKD 2 | 0.004 (0.001) | 0.915 (0.004) | 0.072 (0.004) | 0.008 (0.001) | 0.002 (0.001) | 0.000 (0.000) | 0.000 (0.000) | 0.000 (0.000) |  |
|  | CKD 3a | 0.000 (0.000) | 0.023 (0.001) | 0.910 (0.003) | 0.064 (0.002) | 0.003 (0.001) | 0.000 (0.000) | 0.000 (0.000) | 0.000 (0.000) |  |
|  | CKD 3b | 0.000 (0.000) | 0.001 (0.000) | 0.026 (0.001) | 0.931 (0.002) | 0.041 (0.001) | 0.000 (0.000) | 0.001 (0.000) | 0.000 (0.000) |  |
|  | CKD 4 | 0.000 (0.000) | 0.001 (0.000) | 0.001 (0.000) | 0.028 (0.001) | 0.954 (0.002) | 0.014 (0.001) | 0.002 (0.000) | 0.000 (0.000) |  |
|  | CKD 5 | 0.001 (0.001) | 0.001 (0.001) | 0.001 (0.001) | 0.002 (0.001) | 0.038 (0.005) | 0.910 (0.008) | 0.044 (0.005) | 0.003 (0.002) |  |
|  | Dialysis | 0.000 (0.000) | 0.000 (0.000) | 0.000 (0.000) | 0.000 (0.000) | 0.000 (0.000) | 0.000 (0.000) | 0.998 (0.000) | 0.002 (0.000) | National Dialysis and Transplant Registry MDTR[38] |
|  | Kidney transplant | 0.000 (0.000) | 0.000 (0.000) | 0.000 (0.000) | 0.000 (0.000) | 0.000 (0.000) | 0.000 (0.000) | 0.03 (0.001) | 0.943 (0.001) |  |
