# Supplementary material for: Cost-effectiveness analysis of dapagliflozin for people with chronic kidney disease in Malaysia
Source: PLoS One. 2024 Mar 6;19(3):e0296067. doi: 10.1371/journal.pone.0296067 (PMC10917287; doi:10.1371/journal.pone.0296067)
Supplement: S3 Table — (DOCX) [file pone.0296067.s004.docx]

Supplementary Table 3. Modelled baseline characteristics

|  | Mean (SE) | Reference |
| --- | --- | --- |
| Age (years) | 48.80 (0.32) | Saminathan et al.[10] |
| CKD 1 | 0.25 (0.02) | Saminathan et al.[10] |
| CKD 2 | 0.31 (0.02) | Saminathan et al.[10] |
| CKD 3a | 0.21 (0.00) | Saminathan et al.[10] |
| CKD 3b | 0.21 (0.00) | Saminathan et al.[10] |
| CKD 4 | 0.01 (0.00) | Saminathan et al.[10] |
| CKD 5 (pre-RRT) | 0.01 (0.00) | Saminathan et al.[10] |
| Dialysis | 0.00 (0.00) | Saminathan et al.[10] |
| Transplant | 0.00 (0.00) | Saminathan et al.[10] |
