# Supplementary material for: Cost-effectiveness analysis of dapagliflozin for people with chronic kidney disease in Malaysia
Source: PLoS One. 2024 Mar 6;19(3):e0296067. doi: 10.1371/journal.pone.0296067 (PMC10917287; doi:10.1371/journal.pone.0296067)
Supplement: Attachment — Submitted filename: Cover letter revision.docx [file pone.0296067.s005.docx]

Yee Gary Ang, MBBS MPH
Academic Editor
PLOS ONE

Journal Requirements:

When submitting your revision, we need you to address these additional requirements.

1. Please ensure that your manuscript meets PLOS ONE's style requirements, including those for file naming. The PLOS ONE style templates can be found at
   <https://journals.plos.org/plosone/s/file?id=wjVg/PLOSOne_formatting_sample_main_body.pdf> and 
   <https://journals.plos.org/plosone/s/file?id=ba62/PLOSOne_formatting_sample_title_authors_affiliations.pdf>

We take note of the journal’s requirement. The revised manuscript has been revised to the journals requirement including file naming.

2. Thank you for stating the following financial disclosure: 
"This project is supported by AstraZeneca Malaysia."

Please state what role the funders took in the study.  If the funders had no role, please state: "The funders had no role in study design, data collection and analysis, decision to publish, or preparation of the manuscript." 
If this statement is not correct you must amend it as needed. 
Please include this amended Role of Funder statement in your cover letter; we will change the online submission form on your behalf.

We take note. We have now revised the statement to also include the following.

*This study is funded by AstraZeneca Malaysia. The funder had no role in study design, data collection and analysis, decision to publish, or preparation of the manuscript*

3. Thank you for stating the following in the Acknowledgments Section of your manuscript: 
"The authors thank Dr Hooi Lai Seong for her expert reviews, facilitating the team to validate data inputs, as well as providing editorial assistance. Authors acknowledge this analysis was based on the Dapa-CKD trials published. This study is funded by AstraZeneca Malaysia."

We note that you have provided funding information that is not currently declared in your Funding Statement. However, funding information should not appear in the Acknowledgments section or other areas of your manuscript. We will only publish funding information present in the Funding Statement section of the online submission form. 
Please remove any funding-related text from the manuscript and let us know how you would like to update your Funding Statement. Currently, your Funding Statement reads as follows: 
"This project is supported by AstraZeneca Malaysia."

Please include your amended statements within your cover letter; we will change the online submission form on your behalf.

We take note. This is now included in our revised cover letter and on the online submission to state the following

*This study is funded by AstraZeneca Malaysia. The funder had no role in study design, data collection and analysis, decision to publish, or preparation of the manuscript*

4. We note that you have stated that you will provide repository information for your data at acceptance. Should your manuscript be accepted for publication, we will hold it until you provide the relevant accession numbers or DOIs necessary to access your data. If you wish to make changes to your Data Availability statement, please describe these changes in your cover letter and we will update your Data Availability statement to reflect the information you provide.

We take note. We will not be able to provide the model for other users due to the confidentiality of data but we are happy to provide the information to other people upon reasonable request

5. Please include captions for your Supporting Information files at the end of your manuscript, and update any in-text citations to match accordingly. Please see our Supporting Information guidelines for more information: <http://journals.plos.org/plosone/s/supporting-information>.

We take note and have ensured that the document complies with PLOS One requirement

6. Please review your reference list to ensure that it is complete and correct. If you have cited papers that have been retracted, please include the rationale for doing so in the manuscript text, or remove these references and replace them with relevant current references. Any changes to the reference list should be mentioned in the rebuttal letter that accompanies your revised manuscript. If you need to cite a retracted article, indicate the article’s retracted status in the References list and also include a citation and full reference for the retraction notice.

We take note and have ensured that the document complies with PLOS One requirement

Reviewer #1 had the following comments:

Comment #1. Thank you for the opportunity to review the manuscript entitled "Cost-effectiveness analysis of dapagliflozin for people with chronic kidney disease in Malaysia" The study is well conducted and the manuscript well written. I have a few minor comments.

We thank the reviewer for the encouraging comments and appreciate the efforts and time taken to review our manuscript. We have addressed these comments as delineated below.

Comment #2. It would be preferred if the authors could add under methods that the population in the study was CKD 2-5

We thank the reviewer for the suggestion. We have now edited the sentence as follow

A Markov model was adapted to estimate the economic and clinical benefits of dapagliflozin in people with Stage 2 to 5 CKD

Comment #3. Further under results Change to Dapagliflozin in combination with SoC vs SoC alone

We thank the reviewer for the suggestion. The revised sentence now reads as follow:-

In Malaysia, dapagliflozin in combination with SoC was the dominant intervention compared to SoC alone (RM 81,814 versus RM 85,464; USD19,762 vs USD20,644).
Comment #4. And under introduction, A published Markov model .. I suggest that you omit published here.

We thank the reviewer for the suggestion. We have removed the word published as suggested in our introduction of our abstract. Our revised abstract methods now reads as follow:-

*A Markov model was adapted to estimate the economic and clinical benefits of dapagliflozin in people with Stage 2 to 5 CKD*

Comment #5. In the text of the manuscript you frequently refer to DAPA CKD study, please add ref #17 at each of these referrals.

We thank the reviewer for the suggestion. We have now included the reference to each of these referrals to clarify this.

Comment #6. Page 23 add abbreviation SGLT-2 inhibitor which I also believe should be used as a key word.

We thank the reviewer for the suggestion. We have included SGLT-2 inhibitor as a keyword as proposed.

***Keywords:*** *chronic kidney disease; cost-effectiveness; dapagliflozin; SGLT-2 inhibitor*

Comment #7. Please also check the use of spacebar, sometimes used twice and sometimes missing.

We thank the reviewer for the suggestion. We have checked to ensure that these are consistent throughout our manuscript.

Comment #8. For table 1 and 2 change major hypoglycemia to severe hypoglycemia in line with the international classification.

We thank the reviewer for highlighting this. We have now edited the tables and changed major to severe hypoglycaemia as suggested.

Reviewer #2 had the following comments

Comment #1. In general, the study follows the standard guidelines of conducting cost-effectiveness studies. Some comments should be addressed before acceptance.

We thank the reviewer for spending their invaluable time on our manuscript.

Comment #2.This study was conducted from the perspective of the public sector. Why excluded indirect costs from the analysis?

We take note of the suggestion. We only included the direct costs in our model as the indirect costs such as rent, utilities, administrative expenses are currently unavailable in Malaysia. As such, we are unable to account for the indirect cost into our model. We have now included this very important observation in our revised discussion under study limitations. The revised discussion now read as follow:-

Another limitation was that our model did not include the indirect costs such as rentals, administrative costs or manpower costs associated with treatment in our study due to the lack of publicly available data in Malaysia.

Comment #3.What is the unit for adverse event costs? Is it cost per event or cost per admission? How did authors apply these costs in the Markov model? Was it applied only one cycle or all cycles?

We agree this was unclear. The cost for adverse events represents cost per event which may include hospital admission. The cost was derived from published literature whenever possible. Similar to the model developed by Ewen and colleagues, we accounted for events aligned to those related to DAPA-CKD study, where an individual can experience these events multiple times during the cycle. We have now edited the methods section as follow:-

In the current model, only volume depletion, fractures, diabetic ketoacidosis, severe hypoglycaemic events and amputation were accounted for based upon the results of the DAPA-CKD study. The generalised estimating equation using Poisson distribution was used to estimate the recurrent events including volume depletion, fractures, diabetic ketoacidosis, severe hypoglycaemia and amputations.

Comment #4.Why cost of CKD3a was lower than that of CKD2?

We do note that there appears to be some minor discrepancy in our costs. However, as such costs were not routinely collected in Malaysia, we had to rely on data reported into literature. Nevertheless, these costs data have been validated by our experts who suggest that this may be due to the higher and more aggressive treatment of these individuals.

Comment #5.All costs should be inflated to 2022 instead of 2021.

We agree. We have revised this to the 2022 value as suggested as the GDP values in 2022 were not available at the time of our study. These have now been revised the results throughout our manuscript based upon these new costs and results.

Comment #6.Why did utility of CKD3a, 3b higher than utility of CKD2? In addition, utility of CKD2 is equal to CKD4? Utility of pre-RRT (CKD5) is equal to dialysis? All these data reflect the real situation?

Yes, we agree that these values may not be ideal. However, as described earlier, information related to utility are rarely collected in a Malaysian population. As such, we have utilised the values related to those collected from the DAPA-CKD study instead. This may not be ideal in this situation but represent the best available evidence. We have similarly requested for two experts to verify this who also agreed with our inputs. This is described in our methods as follow:-

In this analysis, the utility inputs for patients CKD 2-5 were derived from the DAPA-CKD[1] study as these unavailable in Malaysia. For utility data on CKD Stage 1, dialysis and transplant, these were sourced from published literature from Malaysia

Comment #7.How did authors apply utility decrement of adverse events? Did author apply to all cycles or specific cycle? Utility decrement of major hypoglycemic events is quite high (0.19). If this adverse event occurs 5-6 times, the initial utility will become almost zero?

We agree this was unclear. The model utilises a Markov state-transition framework where disease progresses through transition between discrete health state characterised by CKD stage with state specific utility decrement and outcome. As the reviewer correctly pointed out, adverse event is considered a transient event, i.e patient remains in the health state for one cycle and then incur event specific cost and utility decrement. These utility decrement only occurs during that cycle and patient will similarly have a utility value for their respective CKD.

Comment #8.Table 3 base-case result: author should display the total cost of each CKD state (if possible). This would help support the conclusion of the study that dapagliflozin arm incurred greater costs of early CKD stage.

We thank the reviewer for the excellent suggestion. We have edited the results to conform with the suggestions of the reviewer as follow:-

Comment #9.Tornado diagram: the range of each parameter and axis title should be clearly specified. The longest bar is secondary/tertiary care population only. What is this parameter related to this study? Is it costs, effects, or utility? The third bar from above is adverse event? Which adverse event author referred to? Which health state utility was in the diagram? Explanation of the result should be added in the text.

We agree this was unclear and can lead to confusion. We have now included and added a description of these results to clarify this. Our revised results now read as follow:-

Results of the model were robust to changes in assumptions, with all estimates falling below the willingness to pay threshold. The tornado plots show how much the results associated with lower and upper parameter values deviate from the mean values result (Fig 2). Larger cost savings were observed if the model only simulated the effects for the 10 years, when the cost of adverse events were varied, and when the health state utility were varied. Probabilistic analysis found that in 99.9% of simulations were below the RM 46,000/QALY (USD11,111/QALY) gained threshold (Fig 3).

Comment #10..Cohort population (Supplement file). Baseline characteristics of DAPA-CKD trial are quite different from those in this study. For DAPA-CKD, the average age is about 61 years. There is no CKD1 at initial (might be few). The majority of the population are CKD3a (31%), CKD3b (44%). However, this study the starting age is 48.80. The majority of the population are CKD1 (25%), CKD2 (31%). Would these differences have an effect on the findings of this study?

We take note of the reviewer’s concern. As highlighted by the reviewer, there are some differences in terms of the population but we believe that the beneficial effects may be applicable to a broader population. We have included these important points in our study discussion under limitations as follow

Nevertheless, there are certain limitations to our model which warrants some discussion. Firstly, the current model does not incorporate the cost savings due to a reduction in heart failure since this information was unavailable in the DAPA-CKD trial. Secondly, our study assumes that the DAPA-CKD population closely mimics the general population in Malaysia. Our analysis assumes that the practice is to treat and start patients early on dapagliflozin treatment where the benefits are more likely to be seen where it can delay disease progression. The analysis represents patients eligible for treatment in Malaysia and thus may not be extrapolated to other countries.

Our model also assumes the broad CKD population, and does not include subgroup analysis such as those with or without diabetes or those with or without heart failure. A higher cost savings is expected especially among those who have diabetes given the greater treatment effects. As the disease transition matrix and utility values were derived from the DAPA-CKD study, this may not reflect the results if the study was implemented in a real-world setting

Reviewer #3 had the following comments.

Comment #1.  Although it is an interesting study , however I have the following comments : -Treatment related adverse events did not include urinary tract infection and genital infection

We agree this is a common and important adverse event for people taking SGLT-2 inhibitors. However, given that these events are considered transient and does not incur significant cost to the healthcare system, we did not account for these in our model. Only Grade 3-5 adverse events were included in our model. To clarify this, we have included this in our revised methods as follow:-

Dapagliflozin, like many other SGLT-2 inhibitors has a well-established safety profile. As such, in the current model, only grades 3 to 5 adverse events that have been found to be significantly different in the DAPA-CKD study[1] were accounted for, including volume depletion, fractures, diabetic ketoacidosis, severe hypoglycaemic events and amputation.

Comment #2 Comparison between diabetic versus non diabetic patients ( it is expected that the annual costs associated with diabetes is high)

We thank the reviewer for the suggestion. However, we were unable to account for these due to the limited information that were available from the DAPA-CKD study which did not report this very important observation. To clarify this, we have included this in the discussion under study limitations as follow:-

Our model also assumes the broad CKD population, and does not include subgroup analysis such as those with or without diabetes or those with or without heart failure. A higher cost savings is expected especially among those who have diabetes given the greater treatment effects.

Comment #3 You should mention in the limitations of the study that there is a difference between a clinical outcome in clinical trials and outcomes observed in real world practice

We agree and thank the reviewer for this very important suggestion. We have included this in our revised discussion section as follow:-

As the disease transition matrix and utility values were derived from the DAPA-CKD study, this may not reflect the results if the study was implemented in a real-world setting.

Comment#4 The results of this cost effective analysis can not be generalized to other countries

We agree. We have revised the discussion section as requested. The revised discussion limitation are as follow

The analysis represents patients eligible for treatment in Malaysia and thus may not be extrapolated to other countries.
