# Supplementary material for: Cost-effectiveness analysis of dapagliflozin for people with chronic kidney disease in Malaysia
Source: PLoS One. 2024 Mar 6;19(3):e0296067. doi: 10.1371/journal.pone.0296067 (PMC10917287; doi:10.1371/journal.pone.0296067)
Supplement: Attachment — Submitted filename: Cover letter revision 2.docx [file pone.0296067.s006.docx]

Yee Gary Ang, MBBS MPH
Academic Editor
PLOS ONE

Dear Prof Ang,

Thank you for kindly considering our article. Based upon the comments from both reviewers, we have made the changes as requested as follow:-

**Reviewer #1 had the following comment. The authors have successfully responded to the comments and the manuscript is now suitable for publication**.

Thank you

**Reviewer #2 had the following comments.**

**Comment # 1. Overall, authors have addressed almost all comments. However, some issues need to be clarified. Authors should use track change in the text for the revised version.**

We take note. The submission includes a track changes as per journal requirement.

**Comment #2. The baseline characteristics of cohort population in DAPA-CKD trial are quite different from those in this study. The majority of cohort population in this study is at the early stage of CKD. The term “closely mimic” in the second limitation might not be appropriate.**

We agree. We have now revised the sentence to read as follow:-

Secondly, as our study population includes mostly people with early stage CKD, it may not reflect the population of those recruited in the DAPA-CKD study.

**Comment #3. Another point is indirect cost. In general, indirect costs are related to productivity loss or time loss. Manpower costs or administrative costs are considered as direct cost in economic analysis.**

We agree. We have now revised the sentence to read as follow

Another limitation was that our model did not include the indirect costs such as productivity loss or transportation costs associated with treatment in our study due to the lack of publicly available data in Malaysia.

We trust that these changes will meet the requirements of the reviewer.

Yours truly

Shaun Lee

On behalf of the authors
