# Supplementary material for: Cost-effectiveness analysis of dapagliflozin for people with chronic kidney disease in Malaysia
Source: PLoS One. 2024 Mar 6;19(3):e0296067. doi: 10.1371/journal.pone.0296067 (PMC10917287; doi:10.1371/journal.pone.0296067)
Supplement: Attachment — Submitted filename: Cover letter revision 3.docx [file pone.0296067.s007.docx]

Yee Gary Ang, MBBS MPH
Academic Editor
PLOS ONE

Dear Prof Ang,

Thank you for kindly considering our article. Based upon the comments from both reviewers, we have made the changes as requested as follow:-

**Reviewer #4 had the following comment**

**Comment #1. The authors stated that “Disease progression was captured using parameters based upon the DAPA-CKD trial” Is there any local study to assure this approach?**

We take note of the reviewer’s comment. In our model, we used the benefits of delay in disease progression using dapagliflozin based upon the data from DAPA-CKD as this information was unavailable locally. Currently, dapagliflozin is only available in Malaysia as a restricted access for people with diabetes at a very limited number. However, disease progression for people with chronic kidney disease was based upon the data from Malaysia’s renal registry. This is described in the methods section as follow.

*Disease progression and mortality*

*Disease progression was captured using parameters based upon the DAPA-CKD trial[17] data supplemented with locally relevant information where applicable[21].*

We have also edited the discussion section as a study limitation as follow:-

*Importantly, as there is a scarcity of information related to the disease transition matrix in Malaysia, data from the DAPA-CKD was used. The disease transition matrix and utility values may not reflect the results if the study was implemented in locally.*

**Comment #2. I really suggest to revise the content of the Introduction. There are numerous repetition, for instance: “Complications associated with CKD include increased risk of all-cause and cardiovascular mortality, kidney disease progression, and fractures” and, “CKD is also associated with an increase in cardiovascular diseases and overall mortality”. OR “has substantial societal and economic impact” and “In addition, it is also associated health-related quality of life (HRQoL) impairments and increase in healthcare costs”. Also, the English needs revision (e.g., “to improved glycaemic control.”**

We take note and thank the reviewer for pointing this out. We have now revised the content of the introduction to be more succinct as follow:-

*Chronic kidney disease (CKD) is estimated to affect 8–16% people globally[1, 2]. In people with CKD, they are associated with an increased risk of all-cause and cardiovascular mortality and fractures.[3-5] This has a far reaching impact, and is associated health-related quality of life (HRQoL) impairments, but also has substantial societal and economic impact.[6] As such, there is significant benefits from halting or delaying CKD progression in the general population.[7-9]*

*In Malaysia, the prevalence of CKD has been increasing over the past decade. The National Health and Morbidity Survey (NHMS) in 2011 reported that the prevalence of CKD was 9.07%.[10] This prevalence increased to 15.48% in the recent prevalence study in 2018.[11] Despite the widespread problem of CKD, treatment options are limited; with only angiotensin-converting enzyme inhibitors (ACEi) and angiotensin receptor blockers (ARB) being the only medications that have consistently shown to slow disease progression.[12-14] Recently, sodium-glucose co-transporter-2 (SGLT2) inhibitor were reported to have cardiovascular and renal benefits in addition to improved glycaemic control.[15, 16]*

**Comment #3. I did not understand why the cost of CKD grade 2 is higher compared to CKD 3a (Table 1)?**

We agree. This information may be confusing but the information was derived from our literature review from a study by Azmi and colleagues which reported a higher cost for CKD grade 2. One plausible reason could be the costing approach used but these were not significantly different between both groups and was considered minor. Nevertheless, we have validated these values with our experts who agreed these are within the usual range of RM 400-500 as per practice in Malaysia. We hope this will help clarify the doubts of the reviewer.

**Comment #4. Please explain in Table 1, what means volume depletion in the current study**

We agree this was unclear. We used the definition of the DAPA-CKD study which stated the following, Volume depletion was defined as an event that resulted in loss of fluid such as dehydration, hypovolemia, or hypotension requiring hospitalisation. To clarify this, we have included the definition as a footnote in Table 1.

**Reviewer #5: I would like to express my sincere gratitude to you for taking the time and effort to address all the comments and feedback provided on your manuscript. Your dedication to refining your work and incorporating the suggested improvements is greatly appreciated. I want to wish you the best of luck as you move forward with the publication process. I have no doubt that your hard work and commitment to your research will lead to a successful and impactful publication.**

We wish to thank the reviewer for the encouraging comments.

We trust that these changes will meet the requirements of the reviewer.

Yours truly

Shaun Lee

On behalf of the authors
